# The Book World of Medicine and Science

**Published:** 1899-10-07

**Authors:** 


### The Book World of Medicine and Science.

How the English Workman Lives. By A German Coal Miner (Ernst Düchershoff). Translated by C. H. d'E. Leppington. (London: P. S. King and Son. 1899. Price 1s.)

This little book is written by a German miner who settled in England. It seems to be a reprint of a number of articles contributed to a Dresden weekly paper describing the life of the Northumberland miners, among whom the author was earning his living, and while it is interesting as giving in simple language a good deal of information—of a very local character, however—concerning the habits and customs of the mining population in the north of England, it is also not without interest as giving us an idea of the views which prevail among German workmen in regard to the position of labour in the Fatherland. The author evidently possesses considerable powers of observation, and it would appear that wherever he worked, whether in Germany or in England, he was able to earn his living without any very great difficulty. But he was an agitator, and got across with the authorities. He often received notice of fines for disorderly conduct, and he was further condemned to seven months' imprisonment for breach of the public peace. Feeling then that under conditions such as these he could not remain in Germany, he came to England, got work at Newcastle, and ultimately wrote this book. But it is clear that his difficulties with the authorities gave him a very strong bias against all things German, while the contrast between the political freedom which he experienced here with the constant police interference in Germany has led him to look through somewhat rose-coloured spectacles on all things English. Writing of the condition of labour in the mining industry, he says, "The position of the English miner is better than that of the German. That the English workmen are not inspired with hatred of the propertied classes results from the fact that the employer is much more humane towards his workpeople than in Germany. I may ask with real astonishment, how is it possible that in Germany miners can be treated so roughly and brutally by the officials? In the pits here there is a spirit of comradeship. Every order is given and carried out in a friendly manner." In describing the relations between the English workman and the religious and benevolent world the author writes strongly against the German system of sick insurance, which forces the worker to employ the doctor appointed by the managers of the fund, and praises the English system of clubs which enables him to select whichever doctor he prefers. As an illustration of the very small idea some people have of the manner in which great hospitals are supported, and of the real meaning of medical charity, it is worth while to note that it never seems to have occurred to this intelligent and observant foreigner that the Royal Infirmary and the dispensary were supported by private charity. He accurately, and evidently from personal experience, describes the working of the out-patient system, beginning with the application to "the pastor or other officer of his church" for a ticket, and ending with the naive admission that after the ticket is out the prescription "can subsequently be made up at any chemist's." He says that both these institutions are most beneficial to the labouring classes, and that "all workmen avail themselves of them in long illnesses, although they may belong to 'doctors' clubs' or friendly societies;" but when it comes to saying whence all these benefits are derived he says, "whether they are supported by the State or by the municipality I do not know. Everything is supplied gratis." Our author even had personal experience of the English poor law, but here again he makes mistakes, looking upon the workhouse as a sort of punishment for cases in which "a man's conduct has been so bad that out-relief is refused." To many, he says, the restraint of the workhouse is so distasteful that they prefer to beg, which, he adds, is permissible in England, "provided that one is singing." It is an interesting book, although obviously biased, and evidently dealing only with the Newcastle district. But the mistakes in some points are sufficiently gross to make us doubt the authenticity of much of the information it supplies.

The Schott Methods of the Treatment of Chronic Diseases of the Heart. By W. Bezly Thorne, M.D., M.R.C.P. Third edition. (London: J. and A. Churchill. 1899. Price 6s.)

The new edition of Dr. Bezly Thorne's well-known work is in several respects an improvement upon those which have preceded it, and may be accepted as an authoritative exposition of the Schott methods as originally practised at Nauheim and now placed at the disposal of heart sufferers in various parts of the world. After a description of Nauheim and its waters separate chapters are given in which the effects of the baths and of the therapeutic movements are described. Then come chapters upon the application of the baths and exercises, and finally some illustrative cases are given. This is not the place to enter into any discussion as to the manner in which the remarkable results obtained are brought about, but we may note that Dr. Bezly Thorne gives the following as being among the effects which have been observed to follow the use of the baths. In three or four days, especially in cases in which the flow of the urine has been scanty, there ensues a free diuresis, which may continue for days or weeks; metabolic change becomes accelerated and improved; deeply-seated organs, more especially the liver and pelvic viscera, are relieved of congestion and partake in the general impulse to functional health; and the heart, relieved of its burden, and contracting fully and without hurry on its contents, derives from an improved coronary circulation materials for the repair of its weakened or damaged tissues. Several diagrams and sphygmographic tracings are given showing the organic change and functional improvement which follow on the use of these baths. It is worth noting here that from the earliest days of this treatment both Professor Schott and his brother have insisted that similar, if not, indeed, identical, effects may be derived from baths artificially prepared so as to resemble the Nauheim waters in their principal constituents. As to the movements a very careful account is given of the chief "gymnastics" employed and of the principles involved in their use, and we are inclined to think that to the practitioner this is likely to be the most useful part of the book, so far as the personal application of the method is concerned. The baths one cannot always obtain, but it is clear that by the use of the exercises alone a very considerable therapeutic effect may be produced by any practitioner who will take the trouble to study the method, and will give time to its application. Taken altogether, the work is one which will be found both interesting and useful.

